# Is the pattern of mandibular asymmetry in mild craniofacial microsomia comparable to non-syndromic class II asymmetry?

**DOI:** 10.1007/s00784-022-04429-6

**Published:** 2022-02-26

**Authors:** Yun-Fang Chen, Shankeeth Vinayahalingam, Stefaan Bergé, Yu-Fang Liao, Thomas Maal, Tong Xi

**Affiliations:** 1 Department of Craniofacial Orthodontics, Chang Gung Memorial Hospital, Taipei, Taiwan; 2 Craniofacial Center, Chang Gung Memorial Hospital, Taoyuan, Taiwan; 3 Craniofacial Research Center, Chang Gung Memorial Hospital, Linkou, Taiwan; 4 Department of Oral and Maxillofacial Surgery, Radboud University Medical Center, Geert Grooteplein 10, 6500 HB Nijmegen, The Netherlands; 5 Department of Craniofacial Orthodontics, Chang Gung Memorial Hospital, Taoyuan, Taiwan; 6 College of Medicine, Chang Gung University, Taoyuan, Taiwan; 7 Radboudumc 3D Lab, Radboud University Medical Center, Nijmegen, The Netherlands

**Keywords:** Craniofacial microsomia, Class II asymmetry, Mandibular asymmetry, Orthognathic surgery planning

## Abstract

**Objectives:**

To compare the characteristics of mandibular asymmetry in patients with unilateral craniofacial microsomia (CFM) and class II asymmetry.

**Materials and methods:**

Pretreatment cone-beam computed tomography of consecutive adults with Pruzansky-Kaban type I and IIA CFM (CFM group) was analyzed by 3D cephalometry. Fourteen mandibular landmarks and two dental landmarks were identified. The mandibular size and positional asymmetry were calculated by using landmark-based linear and volumetric measurements, in terms of asymmetry ratios (affected/non-affected side) and absolute differences (affected − non-affected side). Results were compared with non-syndromic class II with matched severity of chin deviation (Class II group). Statistical analyses included independent *t* test, paired *t* test, chi-square test, and ANOVA.

**Results:**

CFM group (*n*, 21; mean age, 20.4 ± 2.5 years) showed significantly larger size asymmetry in regions of mandibular body, ramus, and condyle compared to Class II group (*n*, 21; mean age, 27.8 ± 5.9 years) (*p* < 0.05). The curvature of mandibular body was asymmetric in CFM. Regarding the positional asymmetry of mandibular body, while a comparable transverse shift and a negligible yaw rotation were found among the two groups, the roll rotation in CFM was significantly greater as well as the occlusal (6.06° vs. 4.17°) and mandibular (7.84° vs. 2.80°) plane cants (*p* < 0.05).

**Conclusions:**

Mild CFM showed significantly more severe size asymmetry and roll rotation in mandible than non-CFM class II asymmetry.

**Clinical relevance:**

To improve the mandibular size and positional asymmetry in CFM, adjunct hard tissue augmentation or reduction in addition to OGS orthodontics with a meticulous roll and yaw planning is compulsory, which is expected to be distinct from treating non-CFM class II asymmetry.

## Introduction

Craniofacial microsomia (CFM) features hypoplastic mandibular structures of the affected side and subsequently a significant facial asymmetry and class II malocclusion [1–3]. Mild CFM (Pruzansky-Kaban type I and IIA) shows mild to moderate mandibular hypoplasia with the presence of a functional temporomandibular joint [4, 5]. In type I and IIA CFM, the extents of the mandibular retrusion and chin deviation can be similar to those of non-syndromic patients with skeletal class II asymmetry. From this point of view, orthognathic surgery (OGS) has been recommended by many clinicians as the standard procedure to substantially treat the dysgnathia and maxillomandibular asymmetry in both groups [5–10].

On account of the aberrant mandibular hypoplasia and malposition, an ideal postsurgical result regarding restoring facial symmetry is often challenging to achieve in non-growing CFM [7, 10–12]. Previous studies revealed that OGS segment repositioning significantly improved the facial midline asymmetry and mandibular retrusion in CFM and non-syndromic class II patients with residual chin deviation of approximately 1 mm and 2.6 mm, respectively [10, 11, 13]. For non-syndromic class II patients, the contour asymmetry improved as well after the conventional OGS approach [13, 14]. However, for CFM patients, a more complex combination of surgical interventions (e.g., different osteotomy designs for affected and non-affected sides, autogenic or alloplastic grafts, patient-specific implant (PSI), bone shaving, myectomy) or a staged treatment protocol was usually proposed in order to obtain a satisfactory treatment outcome for facial contour symmetry [3, 9, 10]. No studies have systematically compared the mandibular morphology between the two groups. A thorough understanding of the similarities and differences in the mandibular shape (revealed by bilateral size differences) and malposition is essential to clarify the necessity of different treatment strategies among patients with mild CFM and asymmetric class II mandibular hypoplasia, and to optimize the treatment protocol for CFM.

The aim of this study was to compare the mandibular morphology affecting the facial asymmetry between subjects with mild CFM and non-syndromic skeletal class II asymmetry. The null hypothesis was that no difference existed in the mandibular characteristics between the two groups.

## Materials and methods

### Study population

Patients older than 16 years with unilateral CFM and skeletal class II asymmetry were enrolled in this retrospective study. CFM group (test group) consisted of consecutive patients who visited the Chang Gung Craniofacial Center between 2010 and 2018 for treatment by using the following inclusion criteria: (1) Pruzansky-Kaban type I or IIA CFM with a deviated chin toward the affected side, (2) availability of cone-beam computed tomography (CBCT) before the orthodontic or orthognathic treatment, (3) absence of craniofacial syndromes other than CFM, and (4) no history of maxillomandibular surgery or trauma. The subjects in Class II group (control group) were consecutively selected from non-syndromic skeletal class II (ANB angle > 4°) patients who visited the same center between 2010 and 2013 for treatment by matching the severity of chin deviation with that of CFM group. The same inclusion criteria were applied to Class II group.

### Image acquisition and cephalometry

**Fig. 1:**
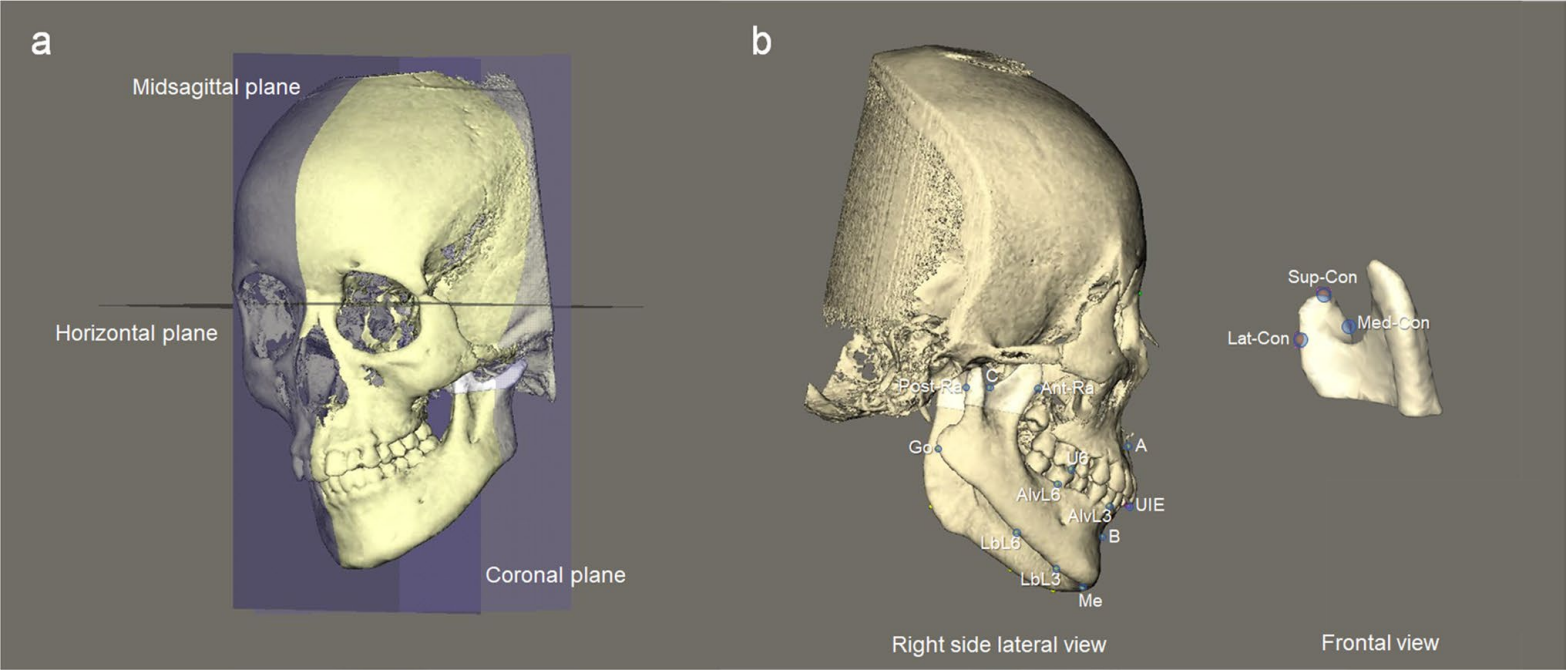
The 3D cephalometric reference frame (**a**). A plane passing through sella and 6-degree below the sella-nasion plane was defined as the horizontal reference plane. A plane passing through sella and nasion and perpendicular to the horizontal reference plane was the midsagittal plane. A plane passing through sella and perpendicular to the horizontal and midsagittal planes was the coronal reference plane. Landmarks used for measurements of mandibular characteristics and facial asymmetry (**b**). Menton (Me), alveolar point at lower canine (AlvL3), alveolar point at lower first molar (AlvL6), mandibular lower border point at lower canine (LbL3), mandibular lower border point at lower first molar (LbL6), gonion (Go), anterior ramal point (Ant-Ra), posterior ramal point (Post-Ra), C-point (C), superior condylar point (Sup-Con), medial condylar point (Med-Con), lateral condylar point (Lat-Con), upper incisal embrasure (UIE), upper first molar (U6). Please refer to Table 1 for the definitions of landmarks

The head and neck of all subjects were scanned in the natural head position using an i-CAT 3D Dental Imaging System (Imaging Sciences International, Hatfield, PA, USA) with parameter settings of 120 kV, 0.4 mm voxel size, 40 s scan time, and 16 cm × 16 cm field of view. CBCT images were stored in the Digital Imaging and Communications in Medicine (DICOM) format and reconstructed to 3D head models using Maxilim® software (Medicim NV, Mechelen, Belgium). Additionally, all the condyle heads were carefully segmented, reconstructed, and integrated with the head models in Maxilim® [15]. The 3D head models were registered in a cephalometric reference frame based on the protocol of Swennen et al., using the landmarks orbitale and porion on the non-affected side, and further completed by the frontozygomatic points, nasion, and sella (Fig. 1) [16, 17]. The affected side was defined as the side to which the chin deviation was pointed to and where CFM was present.

**Fig. 2:**
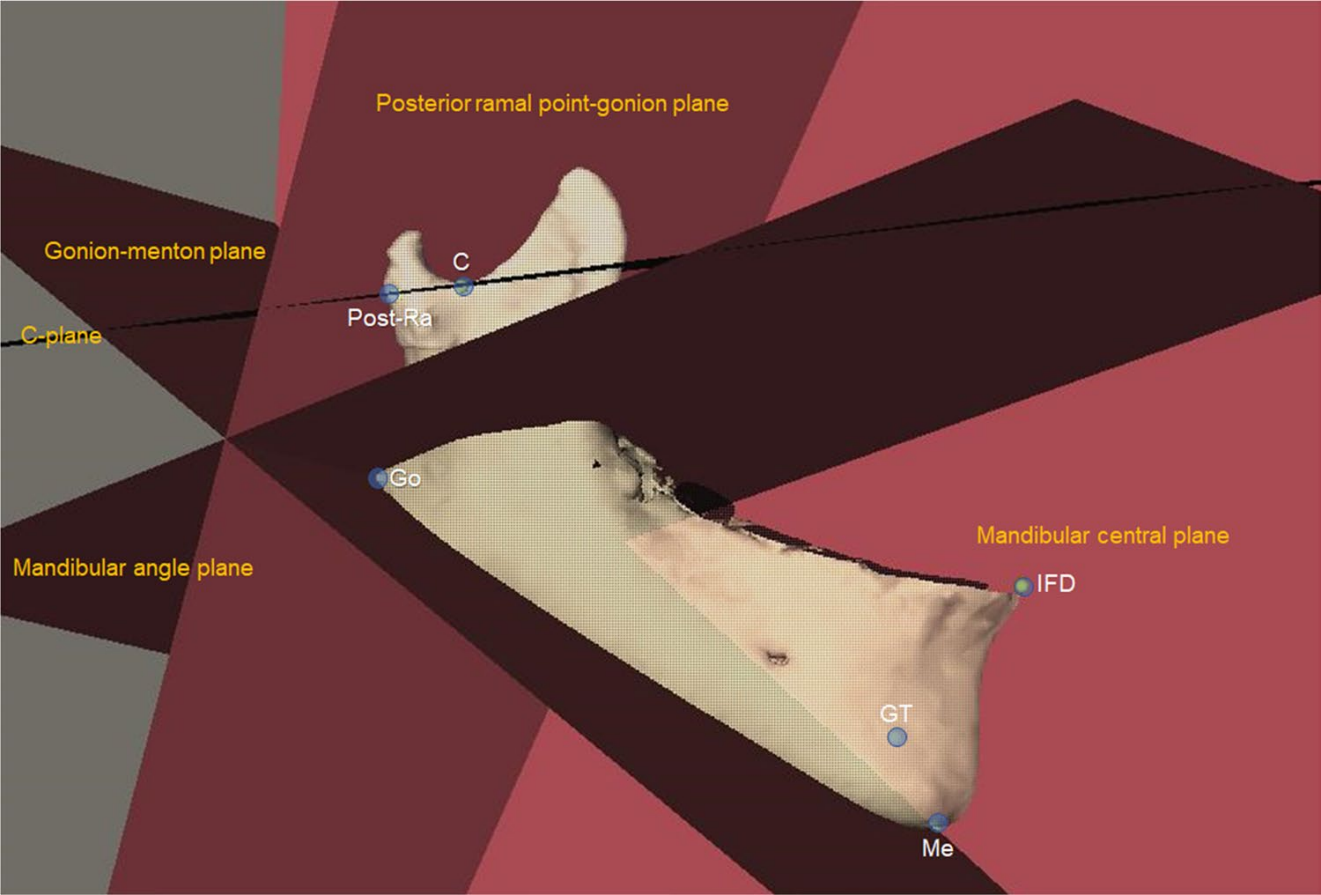
Landmarks and planes used to define the different regions for volumetric measurements. The mandibular central plane was passing through IFD, GT, and Me. The C-plane was passing through the C-point and parallel to the horizontal reference plane. The posterior ramal point-gonion plane was passing through Post-Ra and Go and perpendicular to the mandibular central plane. The gonion-menton plane was passing through Me and Go and perpendicular to the mandibular central plane. The mandibular angle plane was the mid-angular plane between the posterior ramal point-gonion plane and gonion-menton plane. Please refer to Table 1 for the definitions of landmarks

Multiple cephalometric landmarks and planes were used for measurements, which were identified on the 3D head model with the aid of multiplanar views (Tables 1 and 2, Figs. 1 and 2). A positive coordinate value indicated the anterior, inferior, and affected side. Teeth were removed at the level of the alveolar ridge from the 3D mandibular model before volumetric measurements were conducted (Fig. 2). To quantify the size asymmetry of mandible, asymmetry ratios of the mandibular measurements between bilateral sides were calculated (affected side/non-affected side). An asymmetry ratio of 1 indicated perfect symmetry in size. To determine the positional asymmetry of mandible, the difference in coordinates of the affected side minus the non-affected side was recorded.

**Table 1:** Definitions of the 3D cephalometric landmarks and planes

	Symbol	Definition
Landmarks		
Infradentale	IFD	The anterior–superior point on the mandible at its labial contact between the mandibular central incisors
Genial tubercle	GT	The midpoint of the genial tubercle
Menton	Me	The most inferior midpoint of the chin on the outline of the mandibular symphysis
Alveolar point at lower canine^a^	AlvL3	The midpoint of the labial alveolar margin of the mandibular canine
Alveolar point at lower first molar^a^	AlvL6	The midpoint of the buccal alveolar margin of the mandibular first molar
Mandibular lower border point at lower canine^a^	LbL3	The intersection point between the lower border of the mandibular body and a plane passing through Alv-L3 and perpendicular to the mandibular plane
Mandibular lower border point at lower first molar^a^	LbL6	The intersection point between the lower border of the mandibular body and a plane passing through Alv-L6 and perpendicular to the mandibular plane
Gonion^a^	Go	The point at each mandibular angle that is defined by dropping a perpendicular from the intersection point of the tangent lines to the posterior margin of the mandibular ramus and inferior margin of the mandibular body
Anterior ramal point^a^	Ant-Ra	The most anterior point of the mandibular ramus intersecting the C-plane
Posterior ramal point^a^	Post-Ra	The most posterior point of the mandibular ramus intersecting the C-plane
C-point^a^	C	The most caudal point of the sigmoid notch
Superior condylar point^a^	Sup-Con	The most superior point of the condyle
Medial condylar point^a^	Med-Con	The most medial point of the condyle
Lateral condylar point^a^	Lat-Con	The most lateral point of the condyle
Upper incisal embrassure	UIE	The incisal embrasure between the maxillary central incisors
Upper first molar^a^	U6	The mesiobuccal cusp tip of the maxillary first molar
Planes		
Upper occlusal plane		A plane passing through UIE and bilateral U6
Mandibular plane		A plane passing through Me and bilateral Go
Mandibular central plane		A plane passing through IFD, GT, and Me
C-plane^a^		A plane passing through C-point and parallel to the horizontal reference plane
Posterior ramal point-gonion plane^a^		A plane passing through Post-Ra and Go on the same side, and perpendicular to the mandibular central plane
Gonion-menton plane^a^		A plane passing through Me and Go on the same side, and perpendicular to the mandibular central plane
Mandibular angle plane^a^		The mid-angular plane between the posterior ramal point-gonion plane and gonion-menton plane on the same side

^a^Bilateral landmarks or planes

**Table 2:** Definitions of the 3D cephalometric measurements

Measurements	Definition
Linear, mm	
Condylar width	The distance between Lat-Con and Med-Con
Condylar height	The distance between Sup-Con and C-plane
Ramal width	The distance between Ant-Ra and Post-Ra
Ramal height	The distance between C-point and Go
Gonial width	The transverse distance between Me and Go
Anterior mandibular body height	The vertical distance between Alv-L3 and Lb-L3
Posterior mandibular body height	The vertical distance between Alv-L6 and Lb-L6
Mandibular body length	The distance between Me and Go
Chin midline deviation	The distance between Me and the midsagittal plane
x	Perpendicular distance to the midsagittal plane (transverse position of the points)
y	Perpendicular distance to the coronal reference plane (anteroposterior position of the points)
z	Perpendicular distance to the horizontal reference plane (vertical position of the points)
Angular, degrees	
Upper occlusal plane cant	The angle between the upper occlusal plane and the horizontal reference plane projected on the coronal reference plane
Mandibular plane cant	The angle between the mandibular plane and the horizontal reference plane projected on the coronal reference plane
Volumetric, mm^3^	
Condylar volume	The volume of the condylar process cranially to the C-plane
Ramal volume	The volume between the mandibular angle plane and C-plane
Mandibular body volume	The volume between the mandibular central plane and mandibular angle plane

The primary outcome variables were the occlusal plane cant, the mandibular plane cant, and the size and positional asymmetry of the mandible. The primary predictor variable was the type of mandibular asymmetry (CFM vs. class II asymmetry).

To assess the intra-investigator reliability, the CBCT segmentation and measurements were conducted by one investigator (YFC) for 10 randomly chosen patients twice, with an interval of two weeks. To assess the inter-investigator reliability, a second investigator (SV) independently conducted the same process for the same CBCT dataset.

### Statistical analysis

To determine the sample size, the G-Power software (version 3.1.9.7; Franz Faul, University of Kiel, Kiel, Germany) was used. A minimum of 17 subjects per group was estimated based on our previous studies [17, 18] by setting an effect size of 1.0, a significance level of 5% (*p* < 0.05), and a power of 80%.

The Statistical Package for Social Sciences for Windows 24 (SPSS 24, IBM Corp., NY, USA) was used for statistical analyses. All descriptive statistics are presented as mean ± standard deviation. Demographical data of the two groups (CFM and Class II) were compared using an independent *t* test or chi-square test where indicated. The groups were matched by the severity of chin deviation. A paired *t* test was used to compare the difference of CBCT measurements between the affected and non-affected sides. A repeated measures ANOVA with the Bonferroni post hoc test was used to detect significant differences in the CBCT measurements between three locations along the lower border of the mandibular body. Intra-class correlation (absolute, two-way mixed) was calculated to assess the intra- and inter-observer reliability. All statistical tests were two sided, and *p* < 0.05 considered statistically significant.

## Results

### Subject characteristics

Based on the inclusion and exclusion criteria, 21 subjects with unilateral CFM (14 females and 7 males; mean age, 20.4 ± 2.5 years; age range, 17.3–27.0 years) and 21 subjects with non-syndromic class II asymmetry (14 females and 7 males; mean age, 27.8 ± 5.9 years; age range, 19.0–47.0 years) were enrolled. There was no difference in ANB (*p* = 0.580) and SNB (*p* = 0.265) between the groups. However, the SNA of CFM group was significantly lower than Class II group, 78.36° ± 4.32° vs. 82.02° ± 2.73°, respectively (*p* = 0.002) (Table 3).

**Table 3:** Subject characteristics among CFM and Class II groups^a^

	CFM (*n* = 21)	Class II (*n* = 21)	*p*
Age (years)	20.4 ± 2.5	27.8 ± 5.9	< 0.001^b^
Gender: female (*n*)	14	14	1.000^c^
SNA (°)	78.36 ± 4.32	82.02 ± 2.73	0.002^b^
SNB (°)	75.54 ± 5.07	76.99 ± 2.90	0.265^b^
ANB (°)	4.65 ± 2.51	5.04 ± 2.06	0.580^b^
Type I CFM (*n*)	14	-	-
Type IIA CFM (*n*)	7	-	-
Chin deviation (mm)	8.08 ± 4.93	8.72 ± 3.97	0.642^b^

*CFM*, craniofacial microsomia; *Class II*, non-syndromic class II asymmetry

^a^Data are means ± standard deviation except where otherwise indicated

^b^Independent *t* test

^c^Chi-square test

### Measurement reliability

Intra-observer reliability, analyzed by the intraclass correlation coefficient (ICC), was excellent (mean ICC, 0.999; 95%CI, 0.979–1.000). Inter-observer reliability was excellent (mean ICC, 0.999; 95%CI, 0.972–1.000).

### Facial asymmetry

The occlusal plane cant (6.06° ± 3.36° vs. 4.17° ± 2.12°, *p* = 0.040) and mandibular plane cant (7.84° ± 4.10° vs. 2.80° ± 2.90°, *p* < 0.001) were significantly more severe in CFM group than in Class II group (Table 4).

**Table 4:** Comparison of jaw asymmetry between CFM and Class II groups^a^

	CFM (*n* = 21)	Class II (*n* = 21)	*p*
Facial asymmetry			
Upper occlusal plane cant (°)	6.06 ± 3.36	4.17 ± 2.12	0.040
Mandibular plane cant (°)	7.84 ± 4.10	2.80 ± 2.90	< 0.001
Size asymmetry (asymmetry ratio, %)			
Gonial width	74.37 ± 14.01	78.96 ± 9.01	0.330
Mandibular body height at lower canine	91.47 ± 7.31	98.17 ± 6.42	0.003
Mandibular body height at lower first molar	84.30 ± 12.38	99.65 ± 9.83	< 0.001
Mandibular body length	92.28 ± 5.83	95.94 ± 3.43	0.017
Mandibular body volume	81.70 ± 10.97	95.55 ± 7.54	< 0.001
Ramal width	82.06 ± 12.34	99.02 ± 6.58	< 0.001
Ramal height	84.44 ± 14.38	96.42 ± 5.90	0.002
Ramal volume	78.21 ± 26.81	93.30 ± 13.90	0.029
Condylar width	78.88 ± 21.22	75.90 ± 14.02	0.004
Condylar height	53.47 ± 22.79	79.12 ± 14.09	< 0.001
Condylar volume	38.80 ± 23.33	71.92 ± 19.05	< 0.001
Position^b^ asymmetry (asymmetry difference, mm)			
xLbL3	11.60 ± 9.00	13.94 ± 8.22	0.384
yLbL3	1.01 ± 2.98	− 0.04 ± 2.15	0.196
zLbL3	− 6.23 ± 3.15	− 2.23 ± 1.94	< 0.001
xLbL6	7.28 ± 7.32	10.30 ± 6.96	0.190
yLbL6	1.19 ± 5.19	0.34 ± 4.21	0.573
zLbL6	− 10.37 ± 5.28	− 3.86 ± 3.32	< 0.001
xGo	2.36 ± 5.08	5.51 ± 5.52	0.061
yGo	4.38 ± 6.05	− 1.44 ± 3.36	< 0.001
zGo	− 8.41 ± 5.97	− 4.79 ± 3.94	0.026
xSup-Con	0.69 ± 4.87	0.78 ± 4.44	0.950
ySup-Con	− 0.78 ± 6.71	− 0.39 ± 3.39	0.816
zSup-Con	6.26 ± 5.02	1.34 ± 2.08	< 0.001

*CFM*, craniofacial microsomia; *Class II*, non-syndromic class II asymmetry; *A*, affected side; *NA*, non-affected side; *LbL3*, mandibular lower border point at lower canine; *LbL6*, mandibular lower border point at lower first molar; *Go*, gonion; *Sup-Con*, superior condylar point

Asymmetry ratio = affected side/non-affected side

Asymmetry difference = affected side − non-affected side

^a^Data are means ± standard deviation

^b^A positive coordinate value (x, y, z) indicated the anterior, inferior, and affected side of the head

### Mandibular size asymmetry

In CFM group, all the mandibular parameters showed significant size asymmetry (all the values of mandibular size parameters on the affected side were significantly lower than those on the non-affected side). Additionally, the posterior mandibular body height was significantly more asymmetric than the anterior mandibular body height (AlvL6-LbL6 vs. AlvL3-LbL3 = 84.30 ± 12.38% vs. 91.47 ± 7.31%, *p* = 0.002). In Class II group, only gonial width, length, and volume of mandibular body, ramal height, and height and volume of condyle showed significant size asymmetry (Table 5).

**Table 5:** Jaw characteristics of CFM and Class II groups^a^

	CFM (*n* = 21)			Class II (*n* = 21)		
Size	A	NA	*p*	A	NA	*p*
Gonial width (mm)	37.90 ± 5.03	51.70 ± 5.72	< 0.001	41.41 ± 4.21	53.33 ± 4.02	< 0.001
Mandibular body height at lower canine (mm)	23.66 ± 3.51	25.93 ± 3.75	< 0.001	26.17 ± 3.55	26.6.3 ± 2.91	0.234
Mandibular body height at lower first molar (mm)	18.82 ± 4.01	22.52 ± 4.70	< 0.001	22.31 ± 3.50	22.39 ± 3.95	0.856
Mandibular body length (mm)	75.72 ± 6.12	82.08 ± 4.64	< 0.001	81.30 ± 6.00	84.70 ± 4.92	< 0.001
Mandibular body volume (mm^3^)	20,796.98 ± 4584.10	25,664.95 ± 5833.61	< 0.001	24,182.86 ± 4638.41	25,317.66 ± 4690.69	0.012
Ramal width (mm)	29.92 ± 5.52	36.51 ± 4.39	< 0.001	36.62 ± 4.33	37.02 ± 4.05	0.461
Ramal height (mm)	36.75 ± 7.71	43.45 ± 4.54	< 0.001	42.24 ± 7.25	43.75 ± 6.40	0.018
Ramal volume (mm^3^)	6176.64 ± 3220.44	7773.52 ± 2106.82	< 0.003	6766.57 ± 2895.80	7109.76 ± 2227.04	0.189
Condylar width (mm)	15.67 ± 4.17	19.98 ± 2.43	< 0.001	18.70 ± 3.67	19.53 ± 2.67	0.169
Condylar height (mm)	11.39 ± 4.55	21.84 ± 3.40	< 0.001	16.28 ± 3.42	20.77 ± 3.45	< 0.001
Condylar volume (mm^3^)	797.81 ± 424.38	2267.15 ± 905.52	< 0.001	1483.66 ± 466.73	2083.16 ± 484.05	< 0.001
Position^b^	A	NA	*p*	A	NA	*p*
xLbL3 (mm)	22.66 ± 5.70	− 11.06 ± 5.55	< 0.001	23.22 ± 5.65	− 9.28 ± 4.40	< 0.001
yLbL3 (mm)	32.04 ± 11.72	31.03 ± 11.99	0.134	37.28 ± 9.09	37.32 ± 9.50	0.936
zLbL3 (mm)	102.29 ± 8.09	108.51 ± 8.78	< 0.001	107.90 ± 7.44	110.13 ± 7.48	< 0.001
xLbL6 (mm)	35.03 ± 4.65	− 27.75 ± 4.15	< 0.001	37.52 ± 3.75	− 27.22 ± 5.06	< 0.001
yLbL6 (mm)	17.46 ± 9.85	16.28 ± 10.62	0.320	19.07 ± 5.83	18.74 ± 6.99	0.726
zLbL6 (mm)	88.28 ± 8.48	98.65 ± 9.03	< 0.001	95.35 ± 8.76	99.21 ± 7.98	< 0.001
xGo (mm)	45.97 ± 4.68	− 43.61 ± 3.63	0.046	50.13 ± 3.91	− 44.61 ± 4.40	< 0.001
yGo (mm)	− 9.70 ± 6.81	− 13.73 ± 5.98	0.011	− 13.53 ± 4.85	− 12.10 ± 5.06	0.064
zGo (mm)	65.68 ± 10.25	74.08 ± 7.88	< 0.001	71.52 ± 9.80	76.30 ± 8.60	< 0.001
xSup-Con (mm)	51.34 ± 4.36	− 50.65 ± 4.14	0.523	50.86 ± 3.47	− 50.08 ± 3.85	0.429
ySup-Con (mm)	− 9.84 ± 4.46	− 9.06 ± 4.19	0.602	− 10.46 ± 3.95	− 10.07 ± 3.61	0.603
zSup-Con (mm)	20.49 ± 5.56	14.22 ± 3.41	< 0.001	17.90 ± 3.62	16.56 ± 2.86	0.008

*CFM*, craniofacial microsomia; *Class II*, non-syndromic class II asymmetry; *A*, affected side; *NA*, non-affected side; *LbL3*, mandibular lower border point at lower canine; *LbL6*, mandibular lower border point at lower first molar; *Go*, gonion; *Sup-Con*, superior condylar point

^a^Data are means ± standard deviation

^b^A positive coordinate value (x, y, z) indicated the anterior, inferior, and affected side of the head

Compared to Class II group, CFM group showed significantly more severe asymmetry (lower asymmetry ratio) for all the mandibular parameters except gonial width (Table 4).

### Mandibular positional asymmetry

For both groups, LbL3 point, LbL6 point, and gonion on the affected side were significantly positioned laterally and superiorly compared to the non-affected side. For CFM group, gonion on the affected side was significantly positioned anteriorly compared to the non-affected side.

For both groups, Sup-Con point on the affected side was significantly positioned inferiorly compared to the non-affected side (Table 5).

**Fig. 3:**
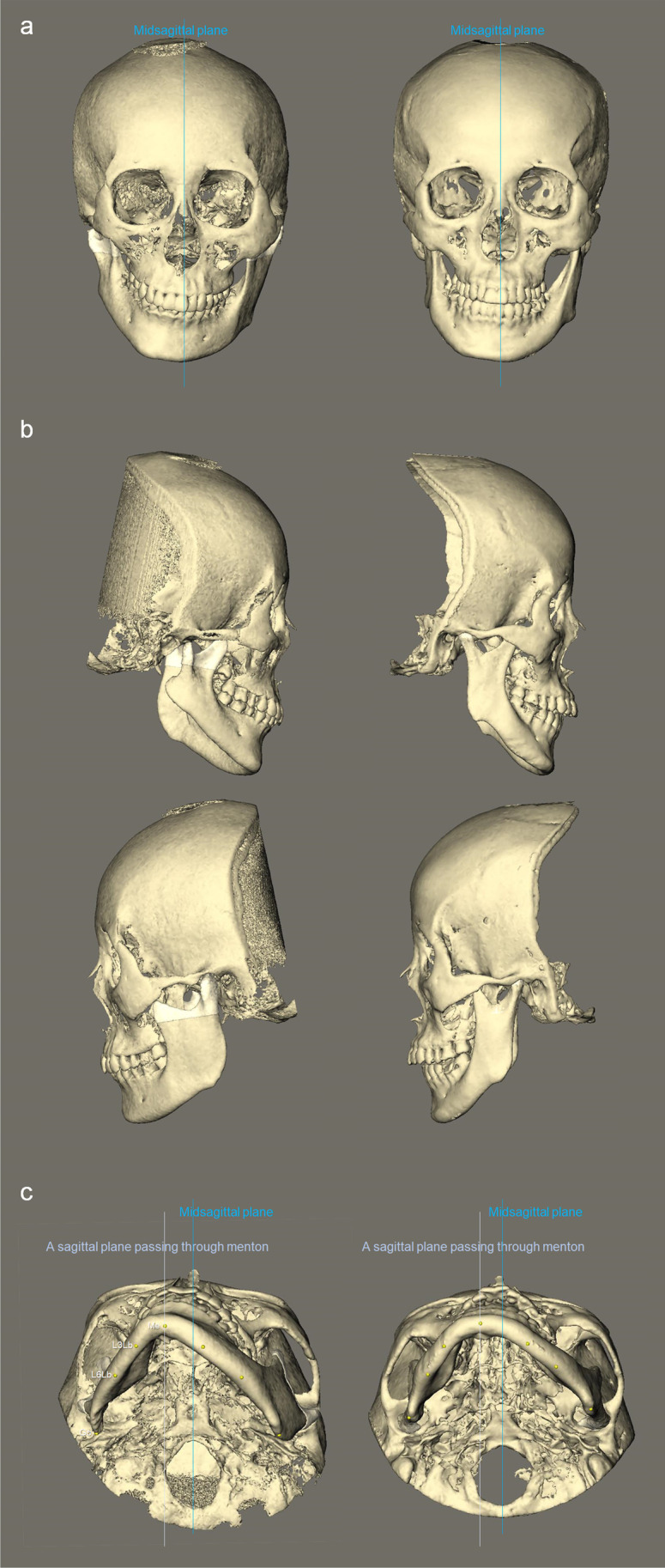
Compared to non-syndromic class II asymmetry (right) with similar extents of chin deviation, skeletal class II discrepancy, and mandibular retrusion, mild CFM (Left) showed: **a** more severe canting of occlusal and mandibular planes; **b** additional maxillary retrusion, and greater height asymmetry in mandibular body, ramus, and condyle; and **c** more severe deficit at the lateral border along the mandibular body (asymmetric shape of body arc), and a sagittally hypoplastic mandibular angle on the CFM-affected side

The repeated measures ANOVA with Bonferroni correction showed that, in relation to the midsagittal plane, there were significant differences between the transverse positional asymmetry of LbL3 point, LbL6 point, and gonion for both groups (CFM, 11.60 ± 9.00 mm vs. 7.28 ± 7.32 mm vs. 2.36 ± 5.08 mm, *p* < 0.01; Class II, 13.94 ± 8.22 mm vs. 10.30 ± 6.96 mm vs. 5.51 ± 5.52 mm, *p* < 0.01), implying more severe side shifting toward the anterior mandible (Fig. 3).

Regarding transverse positional asymmetry at L3Lb point, L6Lb point, gonion, and Sup-Con point, there was no significant difference between the two groups. Regarding the sagittal positional asymmetry, only gonion showed a significant difference between the two groups (CFM vs. Class II = 4.38 ± 6.05 mm vs. − 1.44 ± 3.36 mm, *p* < 0.001). Regarding the vertical positional asymmetry, all the points of LbL3, LbL6, Go, Sup-Con showed significant difference between the two groups (*p* < 0.05) (Table 4).

## Discussion

Clinically, patients with mild CFM (type I and IIA) display similar facial features as non-syndromic class II asymmetry: notable mandibular retrusion and chin deviation. Considering all mandibular regions of interest are present, the CFM-related dysgnathia could be treated by OGS without the need of TMJ reconstruction [7, 9, 10]. Although the commonly known soft tissue deficits in addition to the skeletal hypoplasia in CFM further complicate the facial asymmetry, it has been suggested that soft tissue correction should be conducted after skeletal tissue reconstruction [3, 9, 10]. Even achieving skeletal symmetry is a challenge due to the aberrant shape besides the malposition of the mandible. To the authors’ knowledge, this was the first study to compare the 3D mandibular characteristics between class II asymmetry patients with and without CFM in order to enhance the contemporary OGS treatment strategy and to understand the treatment limitations specifically for mild CFM patients.

The results of the study have rejected the null hypothesis, indicating the presence of a more severe mandibular size asymmetry and vertical positional asymmetry in CFM group than in Class II group, despite a comparable chin deviation.

Compared to non-syndromic class II subjects with similar extent of chin deviation, CFM is characterized by 4 features.

1. CFM subjects displayed significantly more severe occlusal plane cant and mandibular plane cant, and consequently the face was perceived as more asymmetric. Moreover, the canting of the mandibular plane was larger than that of the occlusal plane in CFM group, while the opposite was found in Class II group (Fig. 3a).
2. The mandible of CFM had significantly greater size asymmetry between the affected and non-affected sides, in terms of mandibular, ramal, and condylar heights (Fig. 3b). A remarkable asymmetry in mandibular body height was present particularly in CFM. By contrast, subjects with non-CFM class II asymmetry had relatively symmetric body height, which is accordant with the study of Kim et al. [19]. The reduced ramal height on the chin deviation side is a consistent finding in the literature regardless of the presence of CFM, age, the type of sagittal skeletal discrepancy (class II or III) [8, 20–24], and unsurprisingly, the hypoplastic nature in CFM exaggerated the height discrepancy. The gonial width ratio, on the other hand, was not different between the two groups, indicating similar inward bending of the mandibular angle on the affected side. In CFM group, the asymmetry ratios of width at points of LbL3, LbL6, and gonion were 77.80%, 67.28%, and 74.37%, respectively; in Class II group, 86.60%, 82.34%, and 78.96%. The differences in width asymmetry ratios along the mandibular body implied that the curvature of the mandibular body on the affected side in CFM group resembled a half-V shape (a lateral deficit over the mandibular body), whereas in Class II group, it looked more like a half-U shape (Fig. 3c).
3. CFM group exhibited significant differences in the volume of the mandibular body, ramus, and condyle between the affected and non-affected sides, whereas in Class II group, only the mandibular and condylar volumes were different between the two sides, which were of less severity as well.
4. CFM subjects had significant more maxillary retrusion compared to non-CFM class II asymmetry subjects (Fig. 3b).

Both groups showed a similar transverse shift of the mandible to the affected side without a remarkable yaw rotation. Anteriorly positioned gonion on the affected side was only found among the CFM subjects. This might be the result of unilateral mandibular hypoplasia rather than a yaw rotation of the mandible as there was no significant sagittal discrepancy for bilateral LbL3 and LbL6 points. Also, the significant correlation between the sagittal positional discrepancy of gonion and the length discrepancy of mandibular body in CFM (*r* = 0.508, *p* = 0.019) supported the hypoplastic nature of the mandibular body particularly in CFM.

The four distinctive features between mild CFM and class II asymmetry were the primary findings of the present study. Furthermore, they would also be the fundament to justify different approaches in OGS planning for the two groups of patients.

1. A larger roll correction of the maxilla and mandible is required in mild CFM patients. Moreover, looking into the different patterns of canting in CFM group in contrast to Class II group, the mandibular lower border of the affected side in CFM would remain asymmetric (positioned more cranially) after the occlusal plane cant is surgically leveled (Table 4, Fig. 3). Presurgical orthodontics to decrease the discrepancy between occlusal and mandibular plane cants, or additional hard tissue interventions (reduction, or augmentation with bone graft or PSI) at the mandibular lower border could be considered [11, 12, 25].
2. A shift movement of the maxillomandibular complex toward the non-affected side to center the midline structures will displace the mandibular angle of the non-affected side laterally and that of the affected side medially, deteriorating the posterior mandibular asymmetry, especially in CFM group as the pretreatment transverse positional asymmetry in gonion was less than Class II group (Table 4). A yaw adjustment of the maxillomandibular complex; grinding of the medial side and/or decortication of the lateral side of the proximal segment, or a lingual osteotomy of the distal segment on the non-affected side [26]; or intentional flaring of the proximal segment on the affected side could be considered to reduce the subsequent posterior mandibular asymmetry. The asymmetric mandibular body curvature, or the notable volume deficiency in the mandibular body and ramus on the affected side in CFM would demand further hard or soft tissue interventions (augmentation, reduction, botuline toxine injection, etc.). As the soft tissue envelope on the affected side in CFM is often reduced and compromised, reduction management on the non-affected side with the modest augmentation on the affected side might lead to a more stable outcome than sole augmentation management [10].
3. The retroposition of the maxilla in CFM group would also require a larger maxillary advancement than in Class II group to provide more soft tissue support of the midface and upper lip.

The key in correcting asymmetry of the maxillomandibular complex is the transverse movement of the maxilla and mandible to align the dental and skeletal midlines with the facial midline. As the chin deviation was the same in CFM and Class II groups, a similar transverse movement of the jaws would be required. Promising midline alignment in CFM could be achieved (around only 1 mm of residual chin deviation) as reported in the literature [9, 11], yet the significant bilateral differences in CFM mandibles found in the present study underline the difficulty and the demand for more effort to improve the bilateral asymmetry (cant and contour) in CFM than in cases of non-CFM class II asymmetry.

There are limitations to this current study. First, only hard tissue asymmetry is evaluated in CFM and Class II groups. Soft tissue asymmetry, which also influences the facial appearance, is additionally a core issue in CFM and needs to be inspected in future studies. Moreover, the postsurgical effects of the treatment options described above should be assessed on the soft tissue and hard tissue levels with an adequate follow-up in a clinical comparative study. Lastly, Class II group was significantly older than CFM group. This can be attributed to the very low prevalence of skeletal class II combined with facial asymmetry that is as severe as that of CFM patients. Chew [27] showed a low prevalence of 1.89% for class II asymmetry among 212 orthognathic patients; thus, the prevalence of severe asymmetry can be expected to be lower. Besides, patients with class II asymmetry usually did not have regular visits to our center since their childhood in contrast to CFM patients, and a timely surgical intervention could not be arranged. On the other hand, Pearson’s correlation coefficients showed no significant correlation between age and the extents of facial and mandibular asymmetry in each group, suggesting that the impact of age on skeletal asymmetry might be limited for subjects older than 16 years.

## Conclusions

Mild CFM patients showed more severe size asymmetry in the mandibular body, ramus, and condyle, and displayed a larger roll in the mandibular body compared to patients with non-syndromic skeletal class II asymmetry. Clinicians should be aware of the significant size asymmetry found at the lower (asymmetric body height), posterior (asymmetric angle prominence), and lateral (asymmetric body arc shape) borders of the mandibular body in the treatment of CFM.
